# In vitro template-change PCR to create single crossover libraries: a case study with *B. thuringiensis* Cry2A toxins

**DOI:** 10.1038/srep23536

**Published:** 2016-04-21

**Authors:** Changlong Shu, Jianqiao Zhou, Neil Crickmore, Xianchun Li, Fuping Song, Gemei Liang, Kanglai He, Dafang Huang, Jie Zhang

**Affiliations:** 1 State Key Laboratory for Biology of Plant Diseases and Insect Pests, Institute of Plant Protection, Chinese Academy of Agricultural Sciences, Beijing, 100193, P. R. China; 2 School of Life Sciences, University of Sussex, Falmer, Brighton, UK; 3 Biotechnology Research Institute, Chinese Academy of Agricultural Sciences, Beijing, 100081, P. R. China

## Abstract

During evolution the creation of single crossover chimeras between duplicated paralogous genes is a known process for increasing diversity. Comparing the properties of homologously recombined chimeras with one or two crossovers is also an efficient strategy for analyzing relationships between sequence variation and function. However, no well-developed *in vitro* method has been established to create single-crossover libraries. Here we present an *in vitro* template-change polymerase change reaction that has been developed to enable the production of such libraries. We applied the method to two closely related toxin genes from *B. thuringiensis* and created chimeras with differing properties that can help us understand how these toxins are able to differentiate between insect species.

During evolution, organism speciation is accompanied by duplicated gene divergence (subfunctionalization or neofunctionalization), which can confer environmental adaptation advantages for new species. Diversity can subsequently also arise through the creation of single-crossover chimeras between the resulting paralogues. Recent studies in *Drosophila* have identified a significant number of such chimeric genes that appear to have originated from tandem duplication^1^. This study proposed that qualitative differences in expression, as well as structural differences between the chimeric and parental genes, could enhance rapid evolution. A number of *in vivo* methods^2^,^3^,^4^,^5^ have been developed to create single-crossover chimeric genes, while *in vitro* homologous recombination methods such as DNA shuffling^6^,^7^,^8^, staggered extension process (StEP)^9^,^10^, and other similar methods^11^,^12^,^13^ are best suited to creating multiple-crossover recombination libraries. The three-domain pore-forming Cry toxins of *Bacillus thuringiensis* show evidence of domain swapping through recombination^14^ that has resulted in novel specificities. Previous work has also indicated that the specificity of a Cry toxin can be altered through the creation of *in vitro* chimeras^15^,^16^,^17^,^18^,^19^ making this system ideal for developing an *in vitro* single-crossover method. An advantage of creating these chimeras *in vitro* is that the cell free systems that are increasingly being used in the discovery of therapeutic proteins^20^ can be employed to streamline the downstream screening processes. Here we present an *in vitro* template-change polymerase change reaction developed to enable the production of single-crossover recombination libraries that can mimic those produced *in vivo*.

## Results and Discussion

**Figure 1:**
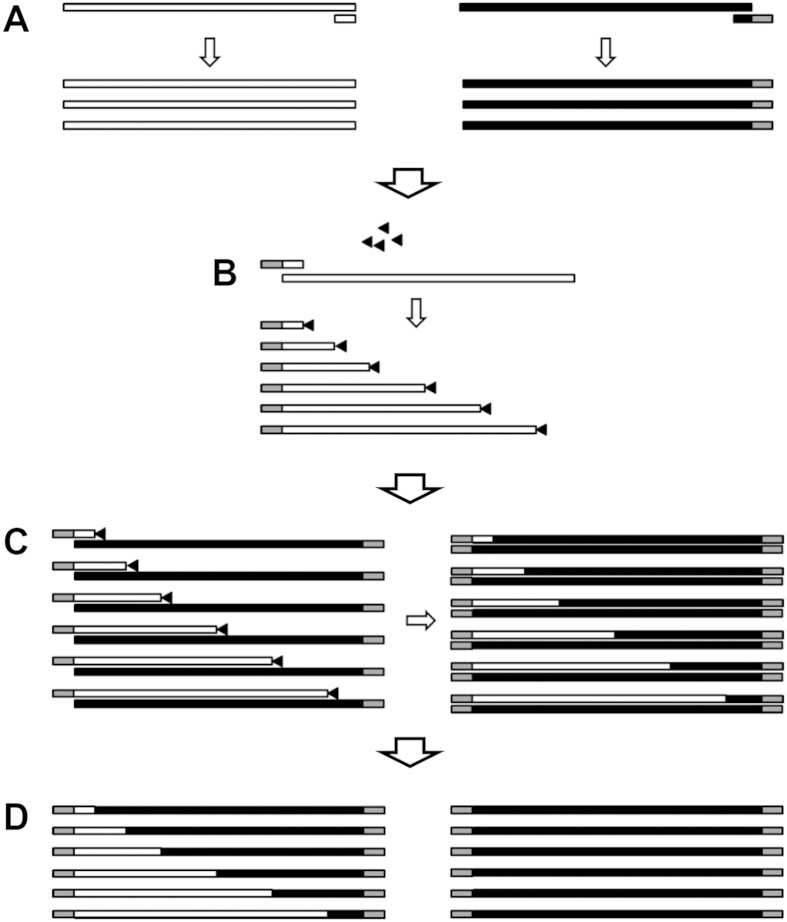
Single-crossover recombination process. Only the forward recombination process is shown. The small triangles represent ddATP molecules, and the gray regions in primers or DNA fragments represent flanking sequences used for selective amplification. (**A**) Generation of reverse ssDNA templates from *cry2Aa* (using primers F and R) and *cry2Ad* (using primers F’ and LR) by asymmetric PCR. (**B**) Generation of randomly terminated *cry2Aa* forward chains using primer LF. (**C**) Re-annealing and extension of *cry2Aa* forward chains using the *cry2Ad* ssDNA template to generate chimeric double-stranded DNA amplicons. (**D**) Use of the flanking sequence primers (SF and SR) for selective amplification and resolution of the chimeras.

To create single-crossover recombinants incompletely extended chains of one of the parent genes are first produced by asymmetric PCR and then annealed to the full length complementary strand of the second parent gene (also produced by asymmetric PCR). The incompletely extended chains are produced through the inclusion of dideoxyATP in the amplification reaction. The frequency of terminations can be controlled by altering the ddATP/dATP ratio. The hybrid formed between the incomplete chain of parent A and the complete chain of parent B is then extended using KOD DNA polymerase. This polymerase has a number of properties that make it suitable for this procedure: it has a 3′ to 5′ exonuclease proofreading activity that is capable of recognizing and removing the ddATP at the 3′ end of the incomplete strand; it is also capable of extending the incomplete strand even if there are one or two un-matched bases at the 3′ end of the incomplete strand. This latter property means that the two parent genes don’t have to have complete identity at the crossover point to produce a chimera. To create crossovers throughout the length of the gene two recombination reactions were performed one using incomplete *cry2Aa* chains primed from the N-terminal end of the gene and the other using incomplete *cry2Ad* chains primed from the C-terminus. The resulting chimeric genes were then amplified using primers that recognized 5′ extensions added to the primers used to generate the templates for the recombination reaction, and which incorporated restriction sites to allow the cloning of the genes into the T7 expression vector pEB for expression in *E. coli* Rosetta (DE3) cells. The above steps are shown diagrammatically in Fig. 1. In this study, two paralogous *Bacillus thuringiensis* toxin genes (*cry2Aa* and *cry2Ad*) were used to test the efficiency of this method. Both genes are 1,899 base pairs (bp) in length and share 89.57% and 86.10% identity at the DNA and protein levels respectively. The Cry2Aa protein is highly toxic to the insects *Ostrinia furnacalis*, *Plutella xylostella*, *Chilo suppressalis*, and *Helicoverpa armigera*^21^, whereas Cry2Ad has little or no toxicity towards these species^22^. In this study, chimeras were designed with a *cry2Aa* N-terminus and a *cry2Ad* C-terminus.

To analyze the recombination efficiency a high-resolution melting (HRM) assay was employed. Two HRM assay primer pairs were designed to amplify the N-terminal (1–157 bp) and C-terminal (1,743–1,899 bp) regions of clones resulting from the recombination reaction. By comparing melting curves, candidates with *cry2Aa* N-terminal and *cry2Ad* C-terminal melting-curve signatures were identified as chimeras. Using this method revealed that 89 clones out of 367 tested were chimeric.

**Figure 2:**
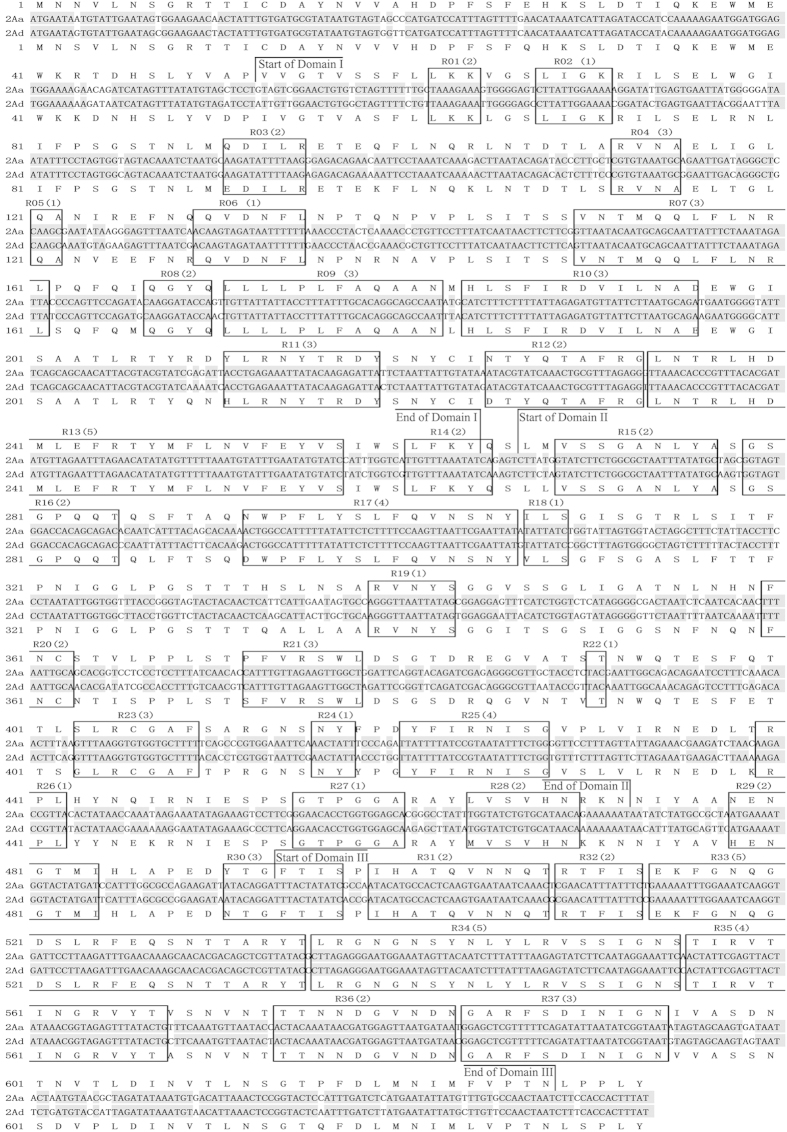
Crossover regions of the generated chimeras relative to a *cry2Aa-cry2Ad* gene alignment. The crossover occurred in a region containing identical DNA sequences between *cry2Aa* and *cry2Ad*, and these are indicated by open rectangles. 2Aa and 2Ad refer to the *cry2Aa* and *cry2Ad* genes respectively. R01 to R37 are the crossover regions of chimeras, the numbers in parentheses means the number of sequenced chimeras with crossovers within that region. Regions of sequence identity are indicated by gray shading and the vertical lines represent the boundaries between the three structural domains.

To analyze the recombination site distribution, these 89 chimeras were sequenced and aligned with the *cry2Aa* and *cry2Ad* gene sequences. The results showed that 37 different chimeras had been produced with crossover points uniformly distributed between bp 158–1,743 of the coding region (Fig. 2). Using the conditions described each recombination reaction was able to create chimeras within a 1 kb region of the priming site for the incomplete chain synthesis.

**Figure 3:**
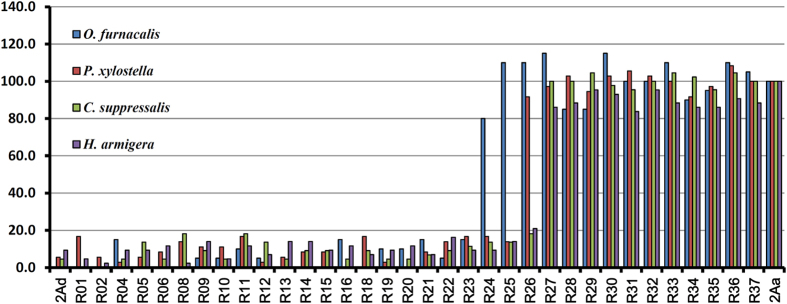
Biological activity of the chimeras. 2Aa and 2Ad refer to the Cry2Aa and Cry2Ad toxins respectively, and R01 through R37 represent the individual chimeras. The y-axis indicates the toxin’s relative toxicity following exposure of the test insect to 50 ppm toxin. The relative activity of each toxin was normalized to the activity of Cry2Aa. The original mortality data are shown in Supplementary Table 2.

Clones representing each of the 37 chimeras were expressed in *E. coli* Rosetta (DE3) cells and the toxic properties of those chimeras that produced a protein of the expected size were evaluated in bioassays (Fig. 3, Supplementary Table 2). A general trend observed was that chimeras R01-R23, which contain up to 409 amino acids from Cry2Aa at their N-termini (and a minimum of 224 amino acids from Cry2Ad at their C-terminus) retain the toxicity profile of Cry2Ad. Chimeras R27-R37 containing 170 or fewer amino acids from Cry2Ad at their C-termini possess the toxicity profile of Cry2Aa. Since the junction between domain I and domain II of the Cry2A toxins is at around amino acid 267, and the junction between domains II and III at around amino acid 474, it can be concluded that substitution of domain I from Cry2Aa does not significantly affect the toxicity or specificity of Cry2Ad. Similarly substitution of domain III from Cry2Ad does not significantly affect the toxicity or specificity of Cry2Aa. Interesting data were obtained with the chimeras in which crossovers took place within domain II indicating that the location of the crossover in which toxicity switched from Cry2Aa-like to Cry2Ad-like was insect specific. For *O. furnacalis* the switch was seen between R23 and R24 whereas for *C. suppressalis* and *H. armigera* the switch was between R26 and R27. For *P. xylostella* the switch was between R25 and R26. There are several reasons why this switch could be insect-specific; it could represent the fact that specific binding of the toxin to the target host cells is mediated by different parts of the toxin in different insects. There is much literature on the involvement of domain II amino acids in the binding of the toxin to host cell receptors^14^,^23^,^24^,^25^ although there is no clear indication that there is a single binding epitope. An alternative explanation is that insect-specific defense mechanisms can differentially affect the efficacy of the toxin. These might include proteases which cleave the toxin at particular locations^26^,^27^ or molecules which sequester the toxin^28^. Such defense mechanisms could differentiate between the chimeras, that is may be active on one but not another. The differences between hybrids either side of a switch location are relatively small, for example between R23 and R24, which differ in *O. furnacalis* toxicity, there are only two amino acids changes at positions 410 and 411. Between R25 and R26 there are three changes (amino acids 430, 433 and 439) as there are between R26 and R27 (443, 446 and 447). Various studies have attempted to define regions of Cry2Aa that define specificity to a number of other insects including the mosquito *Aedes aegypti*^29^, in agreement with our findings they concluded that different regions of domain II of Cry2Aa were crucial for toxicity to different insects.

This example of paralogous gene analysis with *cry2Aa* and *cry2Ad* showed that TC-PCR could efficiently create single-crossover chimeras for analyzing relationships between sequence variation and function. By linkage with cell-free technologies such as ribosome display^20^ or cell line-based technologies, TC-PCR could be broadly used to study structure/function relationships in families of homologous genes. Furthermore, this method can also be applied in directed evolution where one can envision an iterative approach in which successive, low mosaic (ie hybrids with a single crossover), libraries are produced and screened for improved properties.

## Methods

### Generating ssDNA templates of *cry2Aa* and *cry2Ad*

Asymmetric PCR was performed to generate ssDNA copies of *cry2Aa* and *cry2Ad*, using plasmid DNA as template. The PCR was performed in 50 μl reaction mixtures containing 10 nM of each dNTP, 50 nM MgSO_4_, 5 μl 10 × KOD buffer, 0.2 pM forward primer, 20 pM reverse primer and 1.0 unit of KOD DNA polymerase. The reaction conditions were 35 cycles of 94 °C, 1 min; 50 °C (*cry2Aa*)/54 °C (*cry2Ad*), 1 min; and 68 °C, 2.5 min followed by an extension at 68 °C for 7 min. After the addition of 40% deionized formamide the amplicons were purified via a PCR Purification Kit (Axygen) and eluted in 30 μl ddH_2_O. Separate reactions were performed to generate ssDNA copies of both strands for a given gene. All primers used in this study are listed in Supplementary Table 1.

### Generation of incomplete extension chains

To generate incomplete extension chains, a 50 μl PCR reaction mixture was prepared consisting of 1 μl ssDNA template, 12 nM ddATP, 0.4 nM each dNTP, 5 μl 10 × Taq buffer, 20 pM primer, and 2.5 units of *Taq* DNA polymerase. The thermocycling conditions used were as follows: 35 cycles of 94 °C, 1 min; 54 °C, 1 min; and 72 °C, 2.5 min. Following PCR, 40% deionized formamide was added and the products were purified via a PCR Purification Kit (Axygen) and eluted in 25 μl ddH_2_O.

### *cry2Aa* and *cry2Ad* gene recombination

A 50 μl reaction mixture consisting of 10 μl of incomplete extension chains, 1 μl of the corresponding ssDNA full length complementary template from the paralogous gene, 5 μl of 10 × KOD buffer, 10 nM of each dNTP, 50 nM MgSO_4_, 2 μl of dimethyl sulfoxide, and 1.0 unit of KOD DNA polymerase was used to prepare the chimeras. A 5–10 fold excess of incomplete extension chains over template was used. Thermocycling conditions were as follows: 1 cycle of 94 °C, 3 min; 60 °C, 3 min; and 68 °C, 10 min. The recombined products were purified with a PCR Purification Kit (Axygen) and eluted in 30 μl ddH_2_O.

### Selective amplification of *cry2Aa*-*cry2Ad* chimeras

A 50 μl reaction mixture consisting of 1 μl recombined products, 1.5 pM of each selection primer, 5 μl of 10 × KOD buffer, 10 nM of each dNTP, 50 nM MgSO_4_, and 1.0 unit of KOD DNA polymerase was prepared to selectively amplify hybrid genes. PCR was performed using 30 cycles of 94 °C, 1 min; 57 °C, 1 min; and 68 °C, 2.5 min, followed by a final extension step at 68 °C for 7 min. PCR products containing a library of *cry2Aa*-*cry2Ad* chimeras were purified using a DNA Gel Extraction Kit (Axygen) and eluted in 30 μl ddH_2_O.

### Library analysis

The library of *cry2Aa*-*cry2Ad* chimeras was cloned into the pEB cloning/expression vector (Supplementary Fig. 1) using *Bam*HI and *Sal*I, then introduced into JM109 cells and then plated on agar plates containing X-gal. White colonies were picked and used as templates for colony PCR in a 10 μl reaction mixture consisting of 5 μl of 2 × *Taq* mix, a colony template, 1 μl of LC green (Idaho, USA), and 1 μl of the appropriate primer pair (P1 and P1′ for head analysis, or P2 and P2′ for tail analysis). Melting curve analysis^30^,^31^ was performed on the resulting products to distinguish between *cry2Aa* and *cry2Ad* amplicons.

### Expression of *cry2Aa-cry2Ad* chimeras and the parental genes

For *E. coli* expression studies, plasmids encoding parental and chimeric genes were introduced into *E. coli* Rosetta (DE3) competent cells. Transformants were grown at 37 °C in LB medium containing ampicillin (100 mg/l) and chloramphenicol (34 mg/l). After culture densities reached 0.5–1.0 absorbance units at 600 nm, isopropyl thiogalactoside was added to cultures at a final concentration of 0.5 mM, and growth continued at 20 °C. Cells were harvested by centrifugation after 10–12 h of incubation, and pellets were resuspended in 50 mM Tris-HCl (pH 8.0) and sonicated. Harvested proteins were analyzed by sodium dodecyl sulfide-polyacrylamide gel electrophoresis and protein concentrations were determined using the Quantity One gel analysis software package (Bio-Rad, USA).

### Bioassays

Bioassays were performed with *O. furnacalis*, *C. suppressalis*, *H. armigera* using newly hatched larvae at 28 °C and 70% relative humidity. Larvae were fed an artificial diet containing cell lysates with 50 ppm of each toxin protein. For. *P. xylostella* second-instar larvae were fed cabbage leaves dipped into lysates containing 50 ppm of each protein and assayed at 26 °C and 70% relative humidity. Twenty larvae were used for each protein tested, and 3 independent experiments were performed in each case. Mortalities were checked after 7 days for *O. furnacalis*, *C. suppressalis* and *H. armigera* or after 2 days for *P. xylostella*.

## Additional Information

**How to cite this article**: Shu, C. *et al.* In vitro template-change PCR to create single crossover libraries: a case study with *B. thuringiensis* Cry2A toxins. *Sci. Rep.* **6**, 23536; doi: 10.1038/srep23536 (2016).

## Supplementary Material

Supplementary Information
