# Supplementary material for: In vitro template-change PCR to create single crossover libraries: a case study with B. thuringiensis Cry2A toxins
Source: Sci Rep. 2016 Apr 21;6:23536. doi: 10.1038/srep23536 (PMC4838838; doi:10.1038/srep23536)
Supplement: Supplementary Information [file srep23536-s1.pdf]

**Supplementary Information for:**

**In vitro template-change PCR to create single crossover libraries: a case study with *B. thuringiensis* Cry2A toxins.**

Changlong Shu [1], Jianqiao Zhou [1] , Neil Crickmore[2], Xianchun Li [1], Fuping Song [1], Gemei Liang [1], Kanglai He [1], Dafang Huang [3], Jie Zhang [1]*

[1] State Key Laboratory for Biology of Plant Diseases and Insect Pests, Institute of Plant Protection, Chinese Academy of Agricultural Sciences, Beijing, 100193, P. R. China

[2] School of Life Sciences, University of Sussex, Falmer, Brighton, UK

[3] Biotechnology Research Institute, Chinese Academy of Agricultural Sciences, Beijing, 100081, P. R. China

*To whom correspondence should be addressed. (Tel.: 0086-10-62896634, Fax: 0086-10-62812642, E-mail: jzhang@ippcaas.cn).

**Supplementary Figure 1. SDS PAGE analysis of the expressed toxins.**

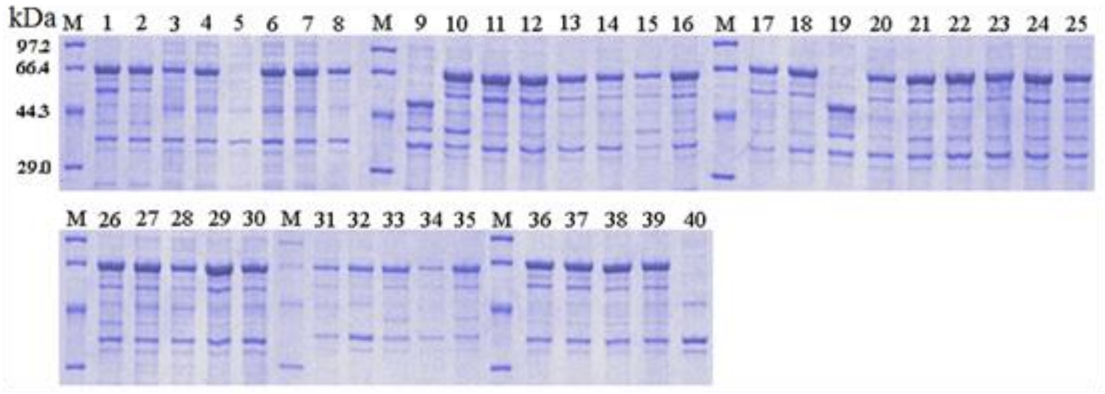


Expression of *cry2Aa*, *cry2Ad* and recombinants in *E. coli* Rosetta. M： Low MW marker; Lane 1: Cry2Aa; Lane 2: Cry2Ad; Lane 40: pEB control; Lanes 3-39: chimeras R01-R37.

**Supplementary Table 1. Sequences of primers used in this study.**

| Name | Sequence |
|---|---|
| F | 5′-ATGAATAATGTATTGAATAGTGGA-3′ |
| R | 5′-ATAAAGTGGTGGAAGATTAGTT-3′ |
| F′ | 5′-ATGAATAGTGTATTGAATAGCGGA-3′ |
| LR | 5′-TTAGCTGGAAACGCGTCGACATAAAGTGGTGAAAGATT-3′ |
| LF | 5′-AACCTTGTAGCGCGGATCCGATGAATAATGTATTGAAT-3′ |
| SR | 5′-TTAGCTGGAAACGCGTCGAC-3′ |
| SF | 5′-AACCTTGTAGCGCGGATCCG-3′ |
| P1 | 5′-ATGAATAATGTATTGAATAGTGG-3′ |
| P1′ | 5′-CAGGAGCTACATATAAACTATGAT-3′ |
| P2 | 5′-TGATAACGGAGCTCGTTT-3′ |
| P2′ | 5′-ATAAAGTGGTGAAAGATTAGTTGG-3′ |

**Supplementary Table 2** Bioassay of the toxicities of the chimeras and parent proteins.

| Samples [a] | *O. furnacalis* | | *P. xylostella* | | *C. suppressalis* | | *H. armigera* | |
|---|---|---|---|---|---|---|---|---|
| | **Mortality** | **SD** | **Mortality** | **SD** | **Mortality** | **SD** | **Mortality** | **SD** |
| Control | 0.00 | 0.00 | 0.00 | 0.58 | 6.67 | 0.58 | 2.22 | 0.58 |
| Cry2Ad | 0.00 | 0.00 | 4.55 | 0.00 | 3.45 | 1.53 | 9.09 | 1.53 |
| R01 | 0.00 | 0.00 | 15.56 | 2.08 | 3.33 | 1.15 | 4.55 | 1.73 |
| R02 | 0.00 | 0.00 | 6.67 | 1.73 | 3.33 | 1.15 | 2.27 | 0.58 |
| R04 | 5.00 | 1.00 | 4.44 | 1.15 | 6.67 | 1.53 | 9.09 | 2.08 |
| R05 | 0.00 | 0.00 | 6.67 | 1.00 | 13.33 | 1.53 | 9.09 | 1.15 |
| R06 | 0.00 | 0.00 | 8.89 | 0.58 | 6.67 | 0.58 | 11.36 | 1.00 |
| R08 | 0.00 | 0.00 | 13.33 | 1.00 | 16.67 | 1.53 | 2.27 | 0.58 |
| R09 | 1.67 | 0.58 | 11.11 | 1.15 | 10.00 | 1.73 | 13.64 | 1.53 |
| R10 | 1.67 | 0.58 | 11.11 | 1.53 | 6.67 | 1.53 | 4.55 | 1.73 |
| R11 | 3.33 | 0.58 | 15.56 | 1.15 | 16.67 | 1.53 | 11.36 | 1.00 |
| R12 | 1.67 | 0.58 | 4.44 | 1.15 | 13.33 | 0.58 | 6.82 | 1.15 |
| R13 | 0.00 | 0.00 | 6.67 | 1.73 | 6.67 | 2.31 | 13.64 | 0.58 |
| R14 | 0.00 | 0.00 | 8.89 | 1.15 | 10.00 | 2.00 | 13.64 | 1.15 |
| R15 | 0.00 | 0.00 | 8.89 | 0.58 | 10.00 | 1.73 | 9.09 | 1.53 |
| R16 | 5.00 | 1.00 | 2.22 | 0.58 | 6.67 | 0.58 | 11.36 | 1.73 |
| R18 | 0.00 | 0.00 | 15.56 | 2.08 | 10.00 | 1.00 | 6.82 | 0.58 |
| R19 | 3.33 | 1.15 | 4.44 | 1.15 | 6.67 | 2.31 | 9.09 | 2.08 |
| R20 | 3.33 | 1.15 | 2.22 | 0.58 | 6.67 | 1.53 | 11.36 | 1.00 |
| R21 | 5.00 | 1.73 | 8.89 | 1.15 | 8.33 | 1.53 | 6.82 | 1.15 |
| R22 | 1.67 | 0.58 | 13.33 | 0.00 | 10.00 | 2.65 | 15.91 | 1.15 |

| | | | | | | | | |
|---|---|---|---|---|---|---|---|---|
| R23 | 5.00 | 1.00 | 15.56 | 2.08 | 11.67 | 2.52 | 9.09 | 1.15 |
| R24 | **26.67** | 1.15 | 15.56 | 1.15 | 13.33 | 1.15 | 9.09 | 2.08 |
| R25 | **36.67** | 0.58 | 13.33 | 1.73 | 13.33 | 0.58 | 13.64 | 0.58 |
| R26 | **36.67** | 1.53 | **75.56** | 0.58 | 16.67 | 2.52 | 20.45 | 2.52 |
| R27 | **38.33** | 1.15 | **80.00** | 0.00 | **76.67** | 1.53 | **84.09** | 1.15 |
| R28 | **28.33** | 3.06 | **82.22** | 0.58 | **76.67** | 0.58 | **86.36** | 1.00 |
| R29 | **28.33** | 0.58 | **77.78** | 0.58 | **80.00** | 1.00 | **93.18** | 1.00 |
| R30 | **38.33** | 1.15 | **82.22** | 1.15 | **75.00** | 0.00 | **90.91** | 1.53 |
| R31 | **33.33** | 1.53 | **86.67** | 0.00 | **73.33** | 0.58 | **81.82** | 1.53 |
| R32 | **33.33** | 3.06 | **84.44** | 0.58 | **76.67** | 0.58 | **93.18** | 1.73 |
| R33 | **36.67** | 1.15 | **82.22** | 0.58 | **80.00** | 1.00 | **86.36** | 1.00 |
| R34 | **30.00** | 2.00 | **75.56** | 0.58 | **78.33** | 1.53 | **84.09** | 0.58 |
| R35 | **31.67** | 1.15 | **80.00** | 0.00 | **73.33** | 1.53 | **84.09** | 2.08 |
| R36 | **36.67** | 3.06 | **88.89** | 1.53 | **80.00** | 0.00 | **88.64** | 0.58 |
| R37 | **35.00** | 1.73 | **82.22** | 0.58 | **76.67** | 0.58 | **86.36** | 1.00 |
| Cry2Aa | **33.33** | 1.15 | **82.22** | 0.58 | **76.67** | 0.58 | **97.73** | 0.58 |

[a] R01 through R37 are chimeras produced for this study. The crossover point for each chimera is shown in Figure 2. The control assay was performed using sample buffer alone. The data show percentage larval mortalities (average of three independent experiments) after exposure to the indicated proteins (50 ppm), mortalities shown in bold refer to activities that were statistically comparable to that observed with Cry2Aa. SD means standard deviation.

**Supplementary Figure 1**

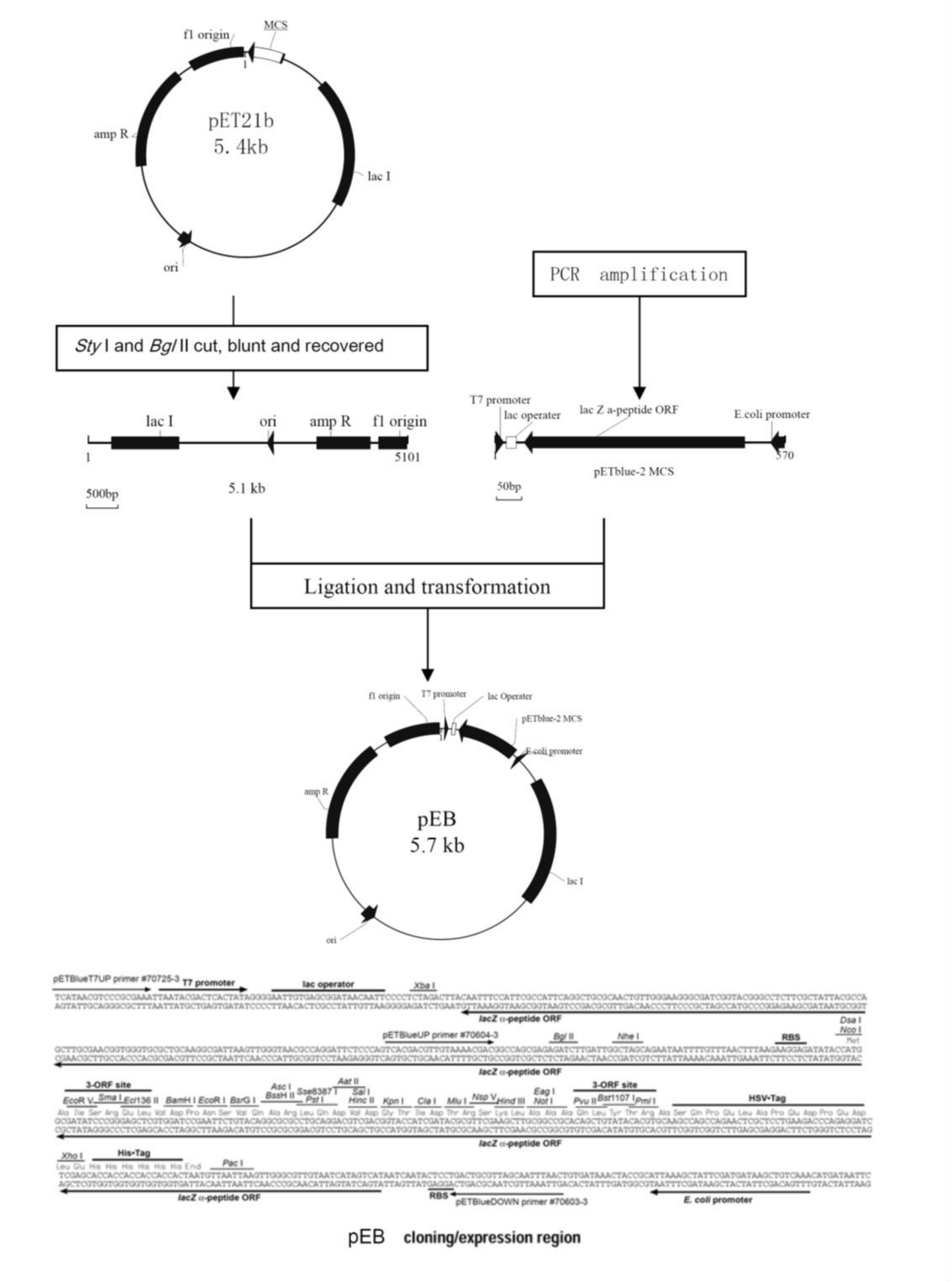


**Supplementary Fig. 1:** Construction of the expression vector. pEB. This was constructed by replacing the expression region of pET21b with that of pETblue-2. The resulting plasmid contains the *lacI* gene and places the T7 promoter directly under the control of the *lac* operator.
